# Association of advanced age with procedural complications and in-hospital outcomes from left atrial appendage occlusion device implantation in patients with atrial fibrillation: insights from the National Inpatient Sample of 36,065 procedures

**DOI:** 10.1007/s10840-022-01266-1

**Published:** 2022-06-22

**Authors:** Muhammad Bilal Munir, Muhammad Zia Khan, Douglas Darden, Zain Ul Abideen Asad, Parnia Abolhassan Choubdar, Mian Tanveer Ud Din, Mohammed Osman, Gagan D. Singh, Uma N. Srivatsa, Sudarshan Balla, Ryan Reeves, Jonathan C. Hsu

**Affiliations:** 1 Section of Electrophysiology, Division of Cardiology, University of California San Diego, La Jolla, San Diego, CA USA; 2 Division of Cardiovascular Medicine, University of California Davis, Sacramento, CA USA; 3 Division of Cardiovascular Medicine, West Virginia University Heart & Vascular Institute, Morgantown, WV USA; 4 Division of Cardiology, University of Oklahoma Health Sciences Center, Oklahoma City, OK USA; 5 Department of Medicine, Allegheny General Hospital, Pittsburgh, PA USA; 6 Division of Cardiovascular Medicine, Oregon Health and Science University, Portland, OR USA; 7 University of California San Diego, 9452 Medical Center Dr., MC7411, La Jolla, San Diego, CA 92037 USA

**Keywords:** Left atrial appendage occlusion, Age, Outcomes, Mortality, Elderly

## Abstract

**Background:**

Age-stratified analyses of atrial fibrillation (AF) patients undergoing percutaneous left atrial appendage occlusion (LAAO) are limited. The purpose of current study was to compare in-hospital outcomes in elderly AF patients (age > 80 years) to a relatively younger cohort (age £ 80 years) after LAAO.

**Methods:**

Data were extracted from National Inpatient Sample for calendar years 2015–2018. LAAO device implantations were identified on the basis of *International Classification of Diseases*, 9th and 10th Revision, Clinical Modification codes of 37.90 and 02L73DK. The outcomes assessed in our study included complications, inpatient mortality, and resource utilization with LAAO.

**Results:**

A total of 36,065 LAAO recipients were included in the final analysis, of which 34.6% (*n*=12,475) were performed on elderly AF patients. Elderly AF patients had a higher prevalence of major complications (6.7% vs. 5.7%, *p* < 0.01) and mortality (0.4% vs. 0.1%, *p* < 0.01) after LAAO device implantation in the crude analysis. After multivariate adjustment of potential confounders, age > 80 years was associated with increased risk of inpatient mortality (adjusted odds ratio [aOR] 4.439, 95% confidence interval [CI] 2.391–8.239) but not major complications (aOR 1.084, 95% CI 0.971–1.211), prolonged length of stay (aOR 0.943, 95% CI 0.88–1.101), or increased hospitalization costs (aOR 0.909, 95% CI 0.865–0.955).

**Conclusion:**

Over 1 in 3 LAAO device implantations occurred in elderly AF patients. After adjusting for potential confounding variables, advanced age was associated with inpatient mortality, but not with other LAAO procedural–related outcomes including major complications, prolonged length of stay, or increased hospitalization costs.

## Introduction  

The prevalence of atrial fibrillation (AF) is projected to increase in the United States (US) largely due to advanced age and concomitant co-morbidities that perpetuate this cardiac dysrhythmia [1, 2]. AF is associated with heightened stroke risk and AF-related strokes tend to be more disabling especially in elderly patients [3, 4]. Additionally, elderly patients have an increased risk of major bleeding when oral anticoagulants are utilized for mitigation of stroke risk in such patients [5, 6]. More recently, left atrial appendage occlusion (LAAO) using a Watchman device has shown promise in reducing the stroke risk as an alternative to OAC therapy [7–9]. LAAO devices can be deemed favorable in elderly patients when compared to OAC therapy due to improved bleeding risk profile. Unfortunately, the landmark randomized trials evaluating the safety and efficacy of LAAO using a Watchman device have limited participation of patients > 80 years old [7, 8]. Additionally, the few retrospective studies evaluating outcomes after LAAO implantation in older patients have shown conflicting results and were not thoroughly adjusted for baseline co-morbidities that commonly occur in this patient population group and could confound such an association with adverse events [10–12]. The aim of the present study was to assess complications and inpatient outcomes while accounting for comorbidities associated with age in elderly AF (> 80 years) patients compared to a younger aged cohort (≤ 80 years) after LAAO implantation.

## Methods

### Data source

Data from National Inpatient Sample (NIS) was used for the purpose of our current study. We analyzed the NIS database from years 2015 to 2018 for LAAO device implantations. The predominant device used in LAAO procedures in our dataset was the first-generation Watchman device since the Watchman FLX and Amulet devices were not approved by the Food and Drug Administration (FDA) during the time frame of our study. The NIS is made possible by a Federal-State-Industry partnership sponsored by the Agency for Healthcare Research and Quality (AHRQ). The NIS is the largest all-payer inpatient healthcare database and is derived from non-Federal hospitals in all States and can be used for computing national estimates of healthcare utilization, costs, and outcomes [13]. The NIS provides discharge weights that are used for estimation of disease and procedure trends nationally. Due to the de-identified nature of NIS dataset, the need for informed consent and Institutional Review Board approval is waived.

### Study population

Watchman device implantations were identified using International Classification of Diseases, 9^th^ Revision, Clinical Modification (ICD-9-CM) and International Classification of Diseases, 10^th^ Revision, Clinical Modification (ICD-10-CM) codes of 37.90 and 02L73DK, respectively, from our dataset. These codes have been extensively utilized in earlier studies for stratification of LAAO devices from administrative datasets [14–16]. Patients younger than 18 years and those with missing demographic data were excluded. Patients were stratified on the basis of age into two sub-groups, patients ≤ 80 years old and patients who were > 80 years of age. Baseline characteristics, procedural complications and inpatient outcomes including mortality (reported as a distinct categorical variable in the dataset), length of stay, and hospitalization costs were compared in Watchman recipients based on age sub-groups. We also analyzed the independent association of age (patients > 80 years versus patients ≤ 80 years) with outcomes of mortality, major complications (defined as composite of pericardial effusion requiring intervention, cardiac arrest, ischemic stroke/transient ischemic attack, hemorrhagic stroke, systemic embolism, myocardial infarction, and peripheral vascular complications which included AV fistula, pseudoaneurysm, access site hematoma, retroperitoneal bleeding, and venous thromboembolism), prolonged hospital stay (defined as length of stay > 1 day), and increased hospitalization cost (median hospitalization cost > 24,327$). For computing hospitalization costs, the cost-to-charge ratio files supplied by Healthcare Cost and Utilization Project were applied to the total hospital charges and adjusted for inflation to December 2018.

### Statistical analysis

Descriptive statistics are presented as frequencies with percentages for categorical variables and as median with interquartile range (IQR) for continuous variables. Baseline characteristics were compared using a Pearson χ^2^ test and Fisher’s exact test for categorical variables and the Kruskal–Wallis *H* test for continuous variables. For crude comparison of procedural complications and in-hospital outcomes among the age groups, the Pearson χ^2^ test was utilized. For assessment of the independent association of age with outcomes including mortality, major complications, length of stay > 1 day, and median hospitalization cost > 24,327$, a single-step multivariable logistic regression model was utilized. Sex, race/ethnicity, CHA_2_DS_2_-VASc score, hospital size, and 29 Elixhauser co-morbidities (heart failure, valvular disease, pulmonary circulation disease, peripheral vascular disease, paralysis, neurological disorders, chronic pulmonary disease, diabetes without complications, diabetes with chronic complications, hypothyroidism, hypertension, renal failure, liver disease, peptic ulcer, acquired immune deficiency syndrome, lymphoma, metastatic cancer, solid tumor without metastasis, collagen vascular disease, coagulopathy, obesity, weight loss, fluid and electrolyte disorders, chronic blood loss anemia, deficiency anemia, alcohol abuse, drug abuse, psychoses, and depression) were used for adjustment of potential confounders. A *p*-value of < 0.05 was considered statistically significant. All statistical analyses were performed using Statistical Package for Social Science (SPSS) version 26 (IBM Corp) and R version 3.6. Because of the complex survey design of NIS, sample weights, strata, and clusters were applied to raw data to generate national estimates.

## Results

We extracted a total of 36,139 LAAO procedures for years 2015 to 2018. Of these, 74 procedures were excluded due to missing demographic patient data, thus yielding a final sample size of 36,065. Baseline characteristics of the study population stratified based on age are shown in Table 1. Out of 36,065 procedures, approximately 12,475 (34.6%) Watchman devices were implanted in patients aged greater than 80 years and 23,590 (65.4%) LAAO Watchman implantations occurred in patients who were aged 80 years or less. Elderly Watchman recipients (patients > 80 years) were more likely to be female, and had a higher burden of specific co-morbidities when compared to a relatively younger cohort of Watchman LAAO recipients (patients ≤ 80 years), including congestive heart failure (35.1% vs. 32.7%, *p* < 0.01), renal failure (25.1% vs. 23.4%, *p* < 0.01), and peripheral vascular disorders (10.9% vs. 9.3%, *p* < 0.01). The prevalence of coronary artery disease (7.7% vs. 7.7%, *p* = 1) and hypertension (85.3% vs. 86%, *p* = 0.06) was similar in both groups.

**Table 1:** Baseline characteristics of the study population of patients undergoing Watchman implantations stratified based on age

Variable no. (%)	Patients > 80 (*n* = 12,475)	Patients ≤ 80 (*n* = 23,590)	*p*-value
Females	5495 (44.0)	9525 (40.4)	< 0.01
Race/ethnicity			
White	10,735 (88.6)	19,340 (84.6)	< 0.01
Black	295 (2.4)	1185 (5.2)	
Hispanic	675 (5.6)	1415 (6.2)	
Asian or Pacific Islander	150 (1.2)	435 (1.9)	
Native American	45 (0.4)	100 (0.4)	
CHA_2_DS_2_-VASc score			
0	0 (0)	130 (0.60)	< 0.01
1	0 (0)	1155 (4.9)	
2	580 (4.6)	4515 (19.1)	
3	3320 (26.6)	7805 (33.1)	
4	4820 (38.6)	6190 (26.2)	
5	2635 (21.1)	2805 (11.9)	
≥ 6	1120 [9]	990 (4.2)	
Median score	4 [3–5]	3 [3–4]	< 0.01
Co-morbidities			
Anemia	2060 (16.5)	3710 (15.7)	0.05
Congestive heart failure	4375 (35.1)	7725 (32.7)	< 0.01
Chronic pulmonary disease	2375 (19.0)	5520 (23.4)	< 0.01
Coagulopathy	520 (4.2)	1000 (4.2)	0.75
Coronary artery disease	965 (7.7)	1825 (7.7)	1
Diabetes	1910 (15.3)	5050 (21.4)	< 0.01
Hypertension	10,635 (85.3)	20,280 (86.0)	0.06
Liver disease	145 (1.2)	795 (3.4)	< 0.01
Obesity	1200 (9.6)	4595 (19.5)	< 0.01
Renal failure	3125 (25.1)	5520 (23.4)	< 0.01
Peripheral vascular disorders	1360 (10.9)	2205 (9.3)	< 0.01
Weight loss	40 (0.3)	120 (0.5)	0.01
Bed size of the hospital			
Small	1515 (12.1)	2390 (10.1)	< 0.01
Medium	2630 (21.1)	5065 (21.5)	
Large	8330 (66.8)	16,135 (68.4)	
Census divisions			
Northeast	1995 (16.0)	3650 (15.5)	< 0.01
Midwest	2705 (21.7)	5155 (21.9)	
South	4495 (36.0)	9405 (39.9)	
West	3280 (26.3)	5380 (22.8)	
Payer			
Medicare	11,820 (94.9)	20,055 (85.2)	< 0.01
Medicaid	40 (0.3)	390 (1.7)	
Private insurance	415 (3.3)	2605 (11.1)	
Self-pay	70 (0.6)	105 (0.4)	
No charge	10 (0.1)	30 (0.1)	
Other	95 (0.8)	360 (1.5)	

Table 2 shows crude in-hospital complications after the implantation of Watchman LAAO devices in our cohort. The prevalence of major complications was higher in elderly AF patients undergoing Watchman implantation when compared to a relatively younger cohort (6.7% vs. 5.7%, *p* < 0.01). This was primarily driven by an increased prevalence of any cardiovascular complication (3.8% vs. 2.8%, *p* < 0.01) and any neurological complication (1.1% vs. 0.7%, *p* < 0.01) in the elderly Watchman cohort. The crude prevalence of additional in-hospital outcomes in AF patients after LAAO Watchman implantation stratified by age subgroups is shown in Table 3. Elderly AF patients undergoing Watchman LAAO implantation experienced in-hospital mortality more frequently when compared to the younger patients (0.4% vs. 0.1%, *p* < 0.01). The prevalence of non-home discharges was also higher in elderly patients when compared to younger patients (10.7% vs. 7.4%, *p* < 0.01).

**Table 2:** Complications in patients undergoing Watchman implantation stratified based on age

Variable no. (%)	Patients > 80 (*n* = 12,475)	Patients ≤ 80 (*n* = 23,590)	*p*-value
Overall complications (%)	1415 (11.3)	2335 (9.9)	< 0.01
Major complications (%)^#^	830 (6.7)	1345 (5.7)	< 0.01
Any cardiovascular complication	470 (3.8)	650 (2.8)	< 0.01
Percutaneous coronary intervention	30 (0.2)	50 (0.2)	0.58
Cardiac arrest	45 (0.4)	25 (0.1)	< 0.01
Heart block	170 (1.4)	170 (0.7)	< 0.01
Pacemaker insertion	55 (0.4)	95 (0.4)	0.22
ST elevation myocardial infarction	< 11 (< 0.09)	25 (0.1)	0.45
Non-ST elevation myocardial infarction	30 (0.2)	85 (0.4)	0.06
Pericardial effusion requiring intervention	220 (1.8)	235 (1.0)	0.001
Pericarditis	30 (0.2)	70 (0.3)	0.33
Cardiogenic shock	45 (0.4)	55 (0.2)	0.03
Any systemic complication	15 (0.1)	40 (0.2)	0.25
Anaphylaxis	0	15 (0.1)	0.01
Arterial embolism	15 (0.1)	15 (0.1)	0.08
Septic shock	0	< 11 (< 0.05)	0.02
Any peripheral vascular complication	185 (1.5)	285 (1.2)	0.02
AV fistula	30 (0.2)	70 (0.3)	0.33
Pseudoaneurysm	50 (0.4)	55 (0.2)	0.01
Access site hematoma	65 (0.5)	80 (0.3)	0.01
Retroperitoneal bleeding	20 (0.2)	15 (0.1)	0.01
Venous thromboembolism	35 (0.3)	85 (0.4)	0.20
Any neurological complication	135 (1.1)	160 (0.7)	< 0.01
Hemorrhagic stroke	40 (0.3)	65 (0.3)	0.45
Ischemic stroke	30 (0.2)	45 (0.2)	0.32
Transient ischemic attack	65 (0.5)	50 (0.2)	< 0.01
Any gastrointestinal or hematological complication	340 (2.7)	615 (2.6)	0.50
Gastrointestinal bleeding	300 (2.4)	580 (2.5)	0.75
Need for blood transfusion	305 (2.4)	430 (1.8)	< 0.01
Any pulmonary complication	330 (2.6)	690 (2.9)	0.12
Respiratory failure	165 (1.3)	400 (1.7)	0.01
Pneumothorax	< 11 (< 0.09)	40 (0.2)	< 0.01
Pleural effusion	70 (0.6)	100 (0.4)	0.07
Pneumonia	55 (0.4)	90 (0.4)	0.4
Need for prolonged ventilator support (> 36 h)	155 (1.2)	350 (1.5)	0.06

**Table 3:** Hospital outcomes and resource utilization in patients undergoing Watchman implantation stratified based on age

Variable no. (%)	Patients > 80 (*n* = 12,475)	Patients ≤ 80 (*n* = 23,590)	*p*-value
Died at discharge	45 (0.4)	25 (0.1)	< 0.01
Discharge disposition			
Home/routine/self-care	11,135 (89.3)	21,385 (92.7)	< 0.01
Non-home discharges	1340 (10.7)	1730 (7.4)	< 0.01
Resource utilization, median (interquartile range)			
Length of stay, days	1 [1–1]	1 [1–1]	0.39
Cost of hospitalization, $	24,295 [18, 915–30, 108]	24,715 [18, 583–30, 934]	0.01

**Fig. 1:**
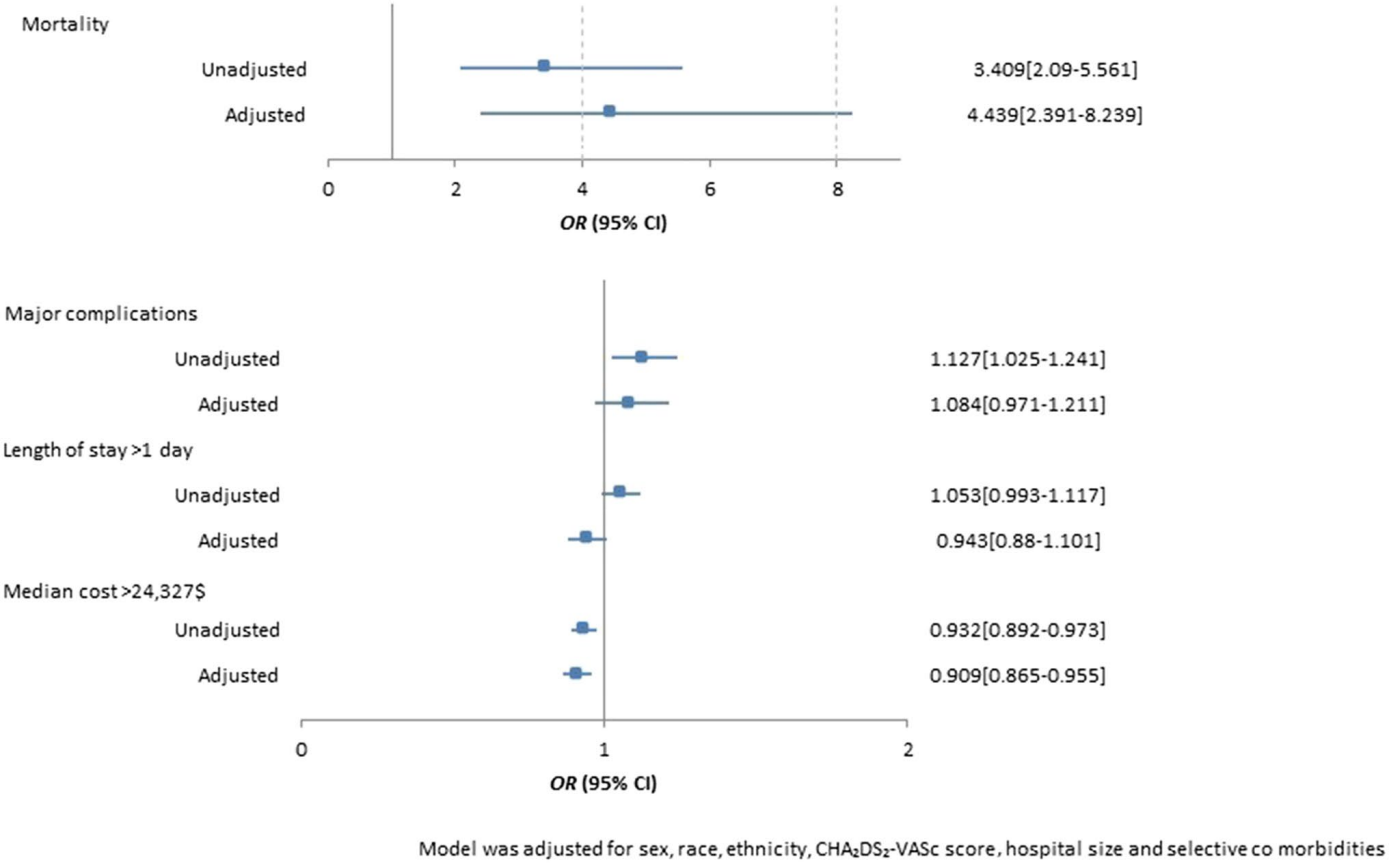
Unadjusted and adjusted associations of age > 80 years with outcomes of mortality, major complications, prolonged length of stay, and increased hospitalization costs in patients undergoing percutaneous left atrial appendage occlusion

To analyze the independent association of age with important outcomes of mortality, major complications, prolonged length of stay (defined as length of stay > 1 day), and hospitalization costs (defined as median cost > 24,327$), a single-step multivariate logistic regression model was created to adjust for potential confounding variables and is shown in Fig. 1. After multivariable adjustment, age > 80 years was found to be an independent predictor of inpatient mortality after Watchman LAAO implantation (adjusted odds ratio [aOR] 4.439, 95% confidence interval [CI] 2.391–8.239). However, after multivariable adjustment, age > 80 years was not associated with major complications (aOR 1.084, 95% CI 0.971–1.211), prolonged length of stay (aOR 0.943, 95% CI 0.88–1.101), or increased hospitalization costs (aOR 0.909, 95% CI 0.865–0.955).

## Discussion

In this large and nationally representative study of AF patients undergoing Watchman LAAO implantation stratified based on age, we report several important findings: (1) Approximately 35% of Watchman LAAO implantations occurred in AF patients > 80 years of age. (2) As expected, elderly patients had a higher burden of some co-morbidities such as congestive heart failure, renal failure, and peripheral vascular disease. (3) The crude prevalence of major complications was higher in elderly AF patients undergoing Watchman LAAO implantation and largely driven by cardiovascular and neurological complications. (4) After multivariable adjustment for potential confounders, age > 80 years was a predictor of inpatient mortality in AF patients undergoing LAAO using a Watchman device but was not associated with other outcomes of major complications, prolonged length of stay, and increased hospitalization costs. These data suggest that although a more frail and comorbid population, elderly AF patients undergoing LAAO implantation appear to have similar risk of adverse events (except in-hospital death and for which the absolute crude difference was small among the two groups, 0.4% vs. 0.1%) when accounting for other comorbidities that are often present with advanced age.

LAAO using an earlier generation Watchman device has shown to be non-inferior to warfarin in the landmark PROTECT AF (Percutaneous closure of the left atrial appendage versus warfarin therapy for prevention of stroke in patients with atrial fibrillation: a randomized non-inferiority trial) and PREVAIL (Prospective randomized evaluation of the Watchman Left Atrial Appendage Closure device in patients with atrial fibrillation versus long-term warfarin therapy) trials [7, 8]. Although no distinct analysis was done for elderly patients in the PROTECT AF and PREVAIL trials, the proportion of patients aged > 75 years was 41% and 52%, respectively, in these trials. Since the comparator arm of these trials constituted subjects tolerating warfarin, advanced age patients (> 80 years) were not well represented in previous randomized controlled trials due to either a prior bleeding event or an increased bleeding risk from underlying comorbidities that are often associated with aging. More recently, newer generation Watchman FLX and Amplatzer Amulet devices are approved by Food and Drug Administration and were studied in the PINNACLE FLX (Primary Outcome Evaluation of a Next Generation Left Atrial Appendage Closure Device) and Amulet IDE (Amplatzer Amulet Left Atrial Appendage Occluder Versus Watchman Device for Stroke Prophylaxis (Amulet IDE): A Randomized, Controlled Trial) trials [17, 18]. The reported prevalence of patients > 75 years of age was 50.5% in the PINNACLE FLX trial. In an age-stratified analysis from the Amulet IDE trail, no differences in the primary efficacy and safety endpoints were noted in patients < 70 years and ≥ 70 years after implantation of Amplatzer Amulet or the Watchman device. Our national cohort of Watchman LAAO recipients demonstrated that approximately 35% of such implantations occurred in elderly AF patients who were more than 80 years, and this patient group was not well represented in the aforementioned trials. The plausible explanation for such higher utilization rate of Watchman LAAO may be that elderly patients > 80 years are more prone to significant bleeding events while on OACs [5, 6] and LAAO provides a viable alternative to such patients for reduction of stroke risk with simultaneous attenuated risk of significant bleeding. Furthermore, our data support the fact that implanting physicians should not hesitate in offering LAAO to eligible elderly patients as it affirmed that these patients do not have an inherent increased risk of major adverse events after LAAO implantation.

Few earlier observational studies have assessed the association of age with procedural related outcomes after LAAO device implantation. In a study of more than 1000 patients from the AMPLATZER cardiac plug multicenter registry, Friexa et al. demonstrated that procedural success was similar at more than 97% in AF patients who were above 75 years of age as compared to the younger cohort after the implantation of the AMPLATZER LAAO device [10]. They also showed no difference in the rate of procedure-related adverse events in their cohort of older and younger AMPLATZER recipients (5.1% vs. 3.2%, *p* = 0.17). In another study of more than 1000 patients undergoing LAAO using a Watchman device, Gonzalez et al. assessed outcomes in elderly patients who were ≥ 85 years old and compared them with a younger cohort of patients [11]. They also found similar procedural success of Watchman implantation in both age groups (98.8% vs. 98.5%, *p* = 0.99). The risk of significant procedure-related adverse events was also similar in both age groups in their cohort of patients undergoing Watchman implantation (2.6% vs. 3.1%, *p* = 0.80). In a more recent study of 6779 LAAO procedures, Sanjoy et al. [12] demonstrated that older patients (≥ 80 years old) experienced a higher prevalence of major adverse events when compared to a younger cohort of LAAO recipients (6% vs. 4.6%, *p* = 0.01). This increased rate of major adverse events in their older cohort was primarily driven by excess magnitude of mortality and cardiovascular complications. Our study expands upon the previous studies, as it is the largest study that adjusted for several comorbid conditions, and also demonstrated increased adjusted risk of mortality in older LAAO device recipients and further depicted that the adjusted risk of other important outcomes such as major complications, prolonged length of stay, and increased hospitalization cost was not different in both the studied age groups. These findings have important implications in the stratification of such patients and can guide implanting physicians with respect to risk/benefit discussion of LAAO in elderly AF patients. Specifically, although elderly patients undergoing LAAO frequently have comorbid conditions, when statistical adjustment for these conditions is performed, the risk of adverse events in this population is not severely elevated except for the risk of mortality.

## Limitations

The results of our current study should be interpreted in the context of following limitations. First, the NIS relies on ICD codes for disease and procedure identification which may be subjected to errors. It should be noted, however, that the NIS has a robust quality control program that minimizes miscoding and ensures data integrity [13]. Second, long-term outcomes cannot be ascertained from the present dataset as NIS does not follow patients longitudinally. Additionally, it is not possible to discern the cause of inpatient mortality from the NIS. Third, NIS does not provide information on anti-coagulation strategy utilized in AF patients and also does not capture any data on frailty which may be sources for potential confounders or reverse causality in our multivariate analysis. Fourth, the NIS only caters to inpatient admissions and does not provide information on outpatient encounters. However, it should be noted that inpatient admission is required for reimbursement of a LAAO device [19]; and hence, our study constitutes a well representative national sample of Watchman implantations in the US in the contemporary period. Fifth, the LAAO device implantations analyzed in this study were first-generation Watchman as newer LAAO devices such as the Watchman FLX and Amulet were not approved by the FDA during our study time period. Additionally, no data on hospital or the operator procedural volume is provided by the NIS.

## Conclusion

Our study showed that a significant proportion of LAAO device implantations (approximately 35%) occurred in elderly AF patients. The crude prevalence of major complications and mortality was higher in elderly AF patients undergoing LAAO device implantation. After multivariate adjustment modeling, age > 80 years was associated with inpatient mortality in our national cohort of LAAO device recipients, but not with other adverse events. Advanced age should not prevent the implanting physicians against offering LAAO device implantation for eligible patients.

## Data Availability

The data that support the finding of this study are available from the first author (MBM) upon reasonable request.
